# Self-awareness of retrosynthesis via chemically inspired contrastive learning for reinforced molecule generation

**DOI:** 10.1093/bib/bbaf185

**Published:** 2025-04-21

**Authors:** Yi Zhang, Jindi Huang, Xinze Li, Wenqi Sun, Nana Zhang, Jiquan Zhang, Tiegen Chen, Ling Wang

**Affiliations:** Guangdong Provincial Key Laboratory of Fermentation and Enzyme Engineering, Joint International Research Laboratory of Synthetic Biology and Medicine, Guangdong Provincial Engineering and Technology Research Center of Biopharmaceuticals, School of Biology and Biological Engineering, South China University of Technology, No. 382 Waihuan East Road, Higher Education Mega Center, Guangzhou 510006, China; Guangdong Provincial Key Laboratory of Fermentation and Enzyme Engineering, Joint International Research Laboratory of Synthetic Biology and Medicine, Guangdong Provincial Engineering and Technology Research Center of Biopharmaceuticals, School of Biology and Biological Engineering, South China University of Technology, No. 382 Waihuan East Road, Higher Education Mega Center, Guangzhou 510006, China; Guangdong Provincial Key Laboratory of Fermentation and Enzyme Engineering, Joint International Research Laboratory of Synthetic Biology and Medicine, Guangdong Provincial Engineering and Technology Research Center of Biopharmaceuticals, School of Biology and Biological Engineering, South China University of Technology, No. 382 Waihuan East Road, Higher Education Mega Center, Guangzhou 510006, China; Guizhou Provincial Engineering Technology Research Center for Chemical Drug R&D, College of Pharmacy, Guizhou Medical University, No. 6 Ankang Avenue, Guian New District, Guiyang 561113, China; Guizhou Provincial Engineering Technology Research Center for Chemical Drug R&D, College of Pharmacy, Guizhou Medical University, No. 6 Ankang Avenue, Guian New District, Guiyang 561113, China; Guizhou Provincial Engineering Technology Research Center for Chemical Drug R&D, College of Pharmacy, Guizhou Medical University, No. 6 Ankang Avenue, Guian New District, Guiyang 561113, China; Zhongshan Institute for Drug Discovery, Shanghai Institute of Materia Medica, Chinese Academy of Sciences, Zhongshan Life Science Park, No. 10 Heqing Road, Tsui Hang New District, Zhongshan 528400, China; Guangdong Provincial Key Laboratory of Fermentation and Enzyme Engineering, Joint International Research Laboratory of Synthetic Biology and Medicine, Guangdong Provincial Engineering and Technology Research Center of Biopharmaceuticals, School of Biology and Biological Engineering, South China University of Technology, No. 382 Waihuan East Road, Higher Education Mega Center, Guangzhou 510006, China

**Keywords:** ATR inhibitor, CDK9 inhibitor, drug discovery, contrastive learning, molecule generation

## Abstract

The recent progress of deep generative models in modeling complex real-world data distributions has enabled the generation of novel compounds with potential therapeutic applications for various diseases. However, most studies fail to optimize the properties of generated molecules from the perspective of the intrinsic nature of chemical reactions. In this work, we propose a novel molecule generation model to overcome the limitation by deep reinforcement learning, in which an agent learns to optimize the properties of molecules initialized with a chemically inspired contrastive pretrained model. We finally assess the generation model by evaluating its ability to generate inhibitors against two prominent therapeutic targets in cancer treatment. Experimental results show that our model could generate 100% valid and novel structures and also exhibits superior performance in generating molecules with fewer structural alerts against several baselines. More importantly, the molecules generated by our proposed model show potent biological activities against ataxia telangiectasia and Rad3-related (ATR) and cyclin-dependent kinase 9 (CDK9) targets in wet-lab experiments.

## Introduction

In the last decades, artificial intelligence has played a substantially more important role in biological and chemical sciences [1–6]. Deep generative models are one of the most powerful ways of modeling or approximating data distributions [7–9], which have been extensively explored in protein sequence generation [10, 11] and molecule design [12]. Recent studies have shown such deep generative models can achieve impressive performance with respect to generating molecules with desired properties [13, 14], which greatly accelerates the traditional drug discovery pipeline [15–17]. Despite the remarkable success of deep generative models for molecule generation, these models often simply learn to optimize the heuristic oracles of molecular properties directly, for example, synthetic accessibility, LogP, and QED scores, failing to explore the chemical properties implied in the chemical reactions [14, 18].

Overall, the main challenges of deep generative models for designing molecules with desirable properties are in three folds: (i) the difficulty of designing valid and diverse molecules. A key challenge in molecular generation is to produce valid and diverse molecules that cover a wide range of chemical space and exhibit a variety of properties [19, 20]. Nonetheless, many of the existing molecular generation algorithms still cannot guarantee the validity and uniqueness of generated molecules [21, 22]. (ii) The difficulty of optimizing molecular properties simply via heuristic oracles. Another challenge in molecule generation is to optimize the desired properties of generated molecules simultaneously, merely guided by heuristic oracles. However, learning to optimize such heuristics from scratch without considering the chemically relevant features is much more complicated for the agent of reinforcement learning [14, 23]. (iii) The difficulty of successfully synthesizing molecules and identifying effective drugs in real-world drug discovery scenarios. A major challenge in molecule generation is ensuring that the generated molecules are synthetically feasible in wet-lab experiments and demonstrate efficacy in a real-world drug discovery and development cycle [24]. Nevertheless, few of the existing models could provide insights for medicinal chemists that help in synthesizing the generated molecules, making it difficult to bridge the gap between computational design and practical laboratory synthesis, let alone ensure their effectiveness in real-world applications.

We propose a novel molecule generation model, pretrained on a chemical reaction dataset using contrastive learning to capture chemical knowledge. The pretrained model is then used to initialize the policy in the reinforced generation process, helping to identify reaction intermediates and reduce reliance on heuristic methods. The molecules are generated fragment by fragment, ensuring chemical validity and diversity. Our key contributions include the following:

- We propose a novel chemically inspired bond augmentation method that is utilized in the contrastive learning stage, in which the chemical bonds that are possible to be broken in the retrosynthetic analysis of a chemical entity are considered as positive pairs.
- In the reinforced molecule generation process, the pretrained model is utilized to initialize the policy by providing molecular representations in the latent space, thus incorporating the chemically relevant properties within the chemical reactions into generated molecules, with a particular focus on the reactive properties of chemical bonds.
- The synthetic pathways are recorded along with the generation process, in which a chemical bond is formed between two fragments in each generation step, inherently embedding synthetic information due to the use of chemically inspired initialization. This provides valuable insights for medicinal chemists during retrosynthetic analysis.
- Experiment results demonstrate that the molecules generated by our model perform well in a range of properties, for example, the validity, uniqueness, medicinal chemistry filters of molecules, and molecule distributions. In addition, the molecules generated have demonstrated potent activity against their targets in wet lab experiments.

## Related work

The remarkable growth spurt of deep generative models, particularly in the form of generative adversarial networks (GANs) [7] and variational autoencoder (VAE) [25], have significantly accelerated the discovery and development of new drugs in the last few years, ultimately accelerating the process of bringing new drugs to market and addressing unmet medical needs. For example, Lim and colleagues proposed a novel approach called conditional VAE (CVAE) that allows for simultaneous control of multiple molecular properties while generating molecules [21]. Popova devised a framework called reinforcement learning for structural evolution (ReLeaSE) [26], which was trained on a dataset of known molecules and their properties and was capable of generating a range of diverse and drug-like molecules with improved properties. De Cao *et al*. adapted GANs to generate molecular graph (MolGAN) [22] and further utilized a reward network and discriminator network to force the generation toward desired chemical properties. Although most of these models could generate molecules with improved properties, few of them are capable of generating molecules with 100% validity, which limits their applications in real-world drug discovery. There is an urgent demand for generating molecules with guaranteed chemical feasibility.

Recently, researchers have attempted to impose molecular validity constraints and property control into the generative models through heuristic oracles. You *et al*. proposed a graph generation policy network (GCPN) [18] and designed an iterative reinforced graph generation process by adding nodes and edges to the intermediate graph, penalizing molecules that violate valency rules. Jin *et al*. proposed junction tree VAE (JT-VAE) [13], which first grouped atoms into substructures such as atoms in a ring and then generated molecules substructure by substructure, thereby ensuring the validity of molecules. In 2021, Yang *et al*. developed a novel fragment-based molecule generation method FREED [12], which decomposed the molecule generation into three distinct steps: determining where to attach a new fragment, selecting which fragment to attach, and deciding where on the new fragment to form a new chemical bond. Goel proposed a reinforcement learning approach MoleGuLAR for generating chemically valid molecules that alternates between optimizing the chemical diversity and synthesizability of molecules [27]. Researchers have recently been endeavoring to impose validity constraints into the generation process by utilizing fragment-based design while utilizing heuristic oracles to optimize the properties of generated molecules. Despite their effectiveness in guaranteeing molecular validity, these approaches typically rely on heuristic oracles to optimize the molecular properties and do not incorporate the domain knowledge within chemical reactions, which makes it difficult for generating molecules with desired properties.

On the other hand, researchers are compelled to work out possible solutions for incorporating the inductive bias through chemical reactions. Bradshaw proposed MOLECULE CHEF [28], a model that mirrored how complex molecules are created in reality. MOLECULE CHEF first maps a multiset of reactants to a distribution over latent space, and a reaction predictor is then fed a multiset of reactants generated from the distribution space to form the final product. In 2020, Horwood and Noutahi proposed a reaction-driven reinforcement learning (REACTOR) framework that leveraged the chemical reactions as an inductive bias, which could explore more efficiently in the synthetically accessible chemical space [29]. Bradshaw put forward a new strategy to guarantee molecular synthesizability, which regarded the synthetic routes as directed acyclic graphs (DAGs) to introduce inductive bias in the generation process [30]. Although these approaches incorporate chemical reactions as an inductive bias to generate molecules with high synthesizability, none of these models are designed to generate molecules while considering the inherent characteristics of chemical reactions from the retrosynthesis viewpoint of medicinal chemists. Herein, we propose a novel bond augmentation method in contrastive learning, which is inspired by the retrosynthetic analysis of chemical products. The chemically relevant features learned during the fine-tuning stage are further utilized to generate molecules with desirable properties while simultaneously accelerating the training process in molecule generation.

## Materials and methods

In this research, we propose a novel molecular representation algorithm based on contrastive learning principles, tailored to retrosynthetic methodology. This algorithm leverages the nature of chemical reactions to generate positive samples during the contrastive learning phase, drawing from retrosynthesis, a key concept in medicinal chemistry. The pretrained contrastive model is then used in the reinforced molecule generation stage, providing molecular embeddings that serve as the foundation for the generation process.

### Model architecture

#### Contrastive learning framework

##### Dataset

**Figure 1:**
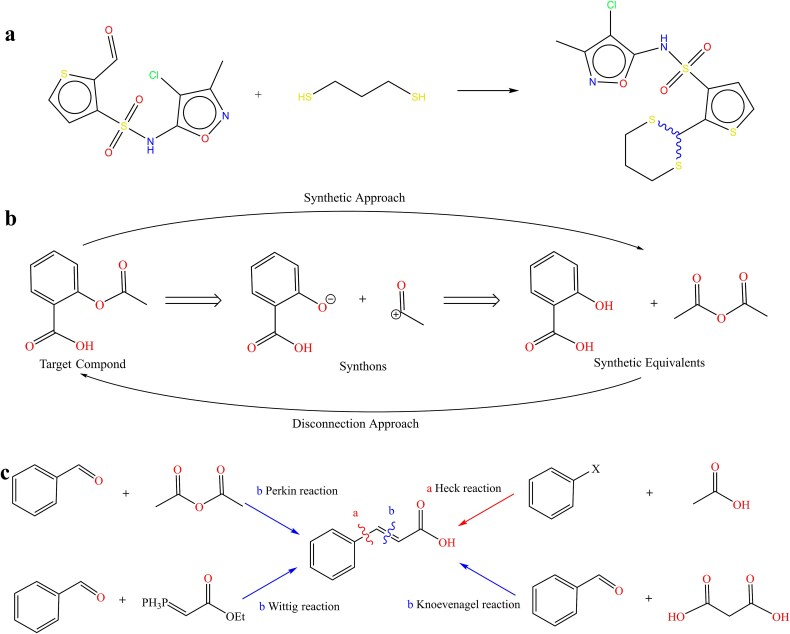
The basic concepts of the RetroCL model. (a) Example of a chemical reaction in USPTO dataset. (b) The retrosynthesis analysis scheme of aspirin. (c) The possible synthesis pathways of cinnamic acid.

In this study, the contrastive pretraining uses the USPTO-MIT [31] and USPTO-50K datasets, widely applied in reaction prediction and retrosynthesis [32, 33]. The USPTO-MIT contains over 1 million reactions, while USPTO-50K, a subset of 50,000 reactions, serves as a benchmark for machine learning models. Figure 1a illustrates a reaction, with wavy bonds marking reaction centers and atom-mapped identifiers showing atom correspondence between reactants and products.

##### Molecule augmentation

This paper focuses on retrosynthetic analysis, a process of tracing a target molecule back to determine viable synthetic steps, enabling synthesis from accessible materials. Formulated by E.J. Corey, retrosynthetic analysis involves breaking the target into synthons and identifying suitable reagents, iterating until laboratory-ready equivalents are found. Each intermediate step leads to a feasible synthesis pathway, reversing the initial disconnection process.

We propose a novel bond augmentation method that treats different disconnected bonds during retrosynthesis as positive augmentations. Using cinnamic acid as an example (Fig. 1c), we demonstrate various reaction pathways, such as Knoevenagel, Heck, Perkin, and Wittig reactions, each forming different bonds. Identifying easily broken or formed bonds, especially carbon–heteroatom and carbon–carbon double bonds, is crucial in retrosynthesis. These bonds, typically weaker and more polar, are key disconnection sites and are easily formed in reactions.

In summary, retrosynthesis breaks down molecules into building blocks, and chemical synthesis forms new molecules by creating stable covalent bonds. Although these bonds differ, they may share common properties in a high-dimensional embedding space, such as electron transitions, bond polarity, and proximity to reactive groups. Our proposed bond augmentation method leverages these shared characteristics to identify synthetic pathways and differentiate between various retrosynthetic routes.

**Figure 2:**
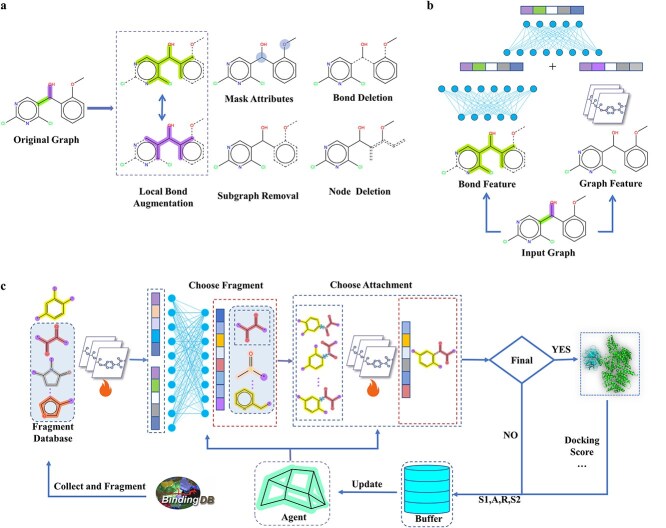
The overview of RetroGEN method. (a) The graph augmentation methods. (b) The hybrid graph and local bond feature encoding network (HGBE) utilized in the contrastive learning model RetroCL. (c) The contrastive learning framework RetroCL. (d) The overall molecule generation process of RetroGEN.

In this work, molecular augmentations are classified into two types: local bond augmentations and graph-level augmentations, as shown in Fig. 2a. Graph augmentations, such as subgraph and attribute masking, bond deletion, and node deletion, are commonly used in graph contrastive learning [34]. Additionally, we introduce a novel local bond augmentation technique, the first to use potential bond breakages in retrosynthesis for augmentations. The highlighted colors, green and purple, in the original molecular graph indicate potential disconnection sites. Local bond augmentation begins with these initial potential breaking bonds, considering the molecular environment within a certain bond range as their feature representation (highlighted in the image). Although these bonds differ, they may share common properties in a high-dimensional space. We treat possible reaction sites as positive views and apply bond augmentations when multiple bonds might break during retrosynthesis. Bond features are defined by local attributes, such as the number of aromatic bonds and nitrogen atoms, as detailed in Supplementary Table S1. These attributes help identify key features that influence bond behavior and reactivity, reflecting characteristics relevant to molecular synthesis and other tasks. These different levels of augmentation consider both the entire molecular graph and the local bond environment, enabling further applications in molecular tasks.

##### Contrastive learning

In this paper, we introduce RetroCL, a novel contrastive learning model that learns a representation function by mapping molecular graphs and local bond features into a latent embedding space. It contrasts positive and negative pairs using large unlabeled data, proving effective in scenarios with limited labeled data. By capturing meaningful patterns, RetroCL enhances generalization and is versatile across domains like image classification [35], language modeling [36], and molecular property prediction [34, 37].

Contrastive learning learns representations by contrasting positive and negative data points. Given a molecular dataset $D=\left\{{x}_1,{x}_2,\dots, {x}_n\right\}$, for each molecular graph ${x}_i$, we generate positive samples ${x}_i^1$ and ${x}_i^2$ through graph-level and bond-level augmentations. Positive pairs share structural context, while negative pairs are distinct samples. These pairs are processed through a hybrid graph and local bond feature encoding network (HGBE) $f(x)$, as shown in Fig. 2b, producing representations ${h}_i^1$ and ${h}_i^2$. The projections $g(x)$ map these to embeddings ${z}_i^1$ and ${z}_i^2$ in embedding space $\epsilon$. The model is trained to maximize similarity between positive pairs and minimize it between negative pairs in $\epsilon$.

Contrastive learning optimizes a feature extractor $f$ and a projection head $g$ to map similar molecular graphs $x$ to closely aligned latent representations $z$ in embedding space. We adopt a loss function akin to the NT-Xent (Normalized Temperature-scaled Cross Entropy Loss), which can be defined as:


(1) \begin{equation*} L=\frac{1}{N}{\sum}_{i=1}^N{l}_i \end{equation*}

where ${l}_i$ in Eq. (1) is what measures the loss between the learned representations of ${x}_i^1$ and ${x}_i^2$. Specifically, it can be denoted as:


(2) \begin{equation*} {\displaystyle \begin{array}{c}{l}_i=-\log \frac{\exp \left(\frac{sim\left({z}_i^1,{z}_i^2\right)}{\tau}\right)}{\sum_{k=1}^N{1}_{k\ne i}\exp \left(\frac{sim\left({z}_i^1,{z}_k^2\right)}{\tau}\right)}-\log \frac{\exp \left(\frac{sim\left({z}_i^2,{z}_i^1\right)}{\tau}\right)}{\sum_{k=1}^N{1}_{k\ne i}\exp \left(\frac{sim\left({z}_i^2,{z}_k^1\right)}{\tau}\right)}\end{array}} \end{equation*}

In Eq. (2), $sim\left(u,v\right)$ represents a similarity measure like cosine similarity, $\tau$ denotes a temperature scaling parameter, and ${1}_{k\ne i}$ is an indicator function, equal to 1 when $k\ne i$ and $N$is the number of samples in the batch.

In this research, we employ multilevel strong augmentations of the molecular data, which has been proven effective in improving the quality of the learned representations [38, 39]. For example, in computer vision, applying strong augmentations like random cropping, rotation, and color jittering, or automated strategies, helps models recognize objects from various angles and lighting conditions [38, 40]. We will demonstrate the effectiveness of the proposed augmentations in the related downstream tasks.

#### Reinforced molecule generation

##### Reinforced molecule generation

In our study, as shown in Fig. 2d, we use reinforcement learning to optimize the generated molecules. The model, RetroGEN, follows a fragment-based design process, consisting of two steps: (i) selecting which fragment to add and (ii) determining which bond to form. Starting with a scaffold, such as a benzene ring, fragments are added from the library based on reinforcement learning to maximize the cumulative reward and final score (e.g., synthetic accessibility score and synthetic accessibility score). The addition of each fragment introduces multiple bonding strategies, which significantly impact the properties of the generated molecules.

##### Reinforced molecule initialization with pretrained encoder

The pretrained molecular graph and local bond embedding model are used to embed intermediate states in the reinforcement learning generation process. These embeddings serve as initial representations, enabling the agent to focus on key molecular features, particularly in data-limited scenarios. The policy network is initialized with expert strategies from the pretrained model, emphasizing likely broken bonds during retrosynthesis. The agent forms bonds between fragments to maximize rewards, including synthetic accessibility, guiding efficient synthesis for medicinal chemists. While some reactions in the USPTO dataset may be complex, we treat them as simple and efficient pathways for this study. Experimental details are provided in the supplementary materials.

## Results

In this section, extensive experiments are conducted to validate the effectiveness of our proposed method by answering the following questions:

***Question I Does contrastive pretraining RetroCL learn about important features related to synthesis within chemical reactions?***

We propose a novel molecular bond augmentation strategy in contrastive learning to capture key structural and chemical features related to molecular synthesis, improving predictions of bond breakage in reactions. In the fine-tuning stage, we will verify whether the model has learned bond-breaking features for retrosynthesis.

***Question II Can the contrastive learning model RetroCL be applied in molecule generation?***

We initialize the reinforced molecule generation model with the contrastive pretrained model RetroCL, which guides fragment selection and attachment strategies based on designed rewards, thereby influencing molecular properties. We then explore its application in molecule generation and analyze the distributions of the generated molecules’ properties.

***Question III Does the proposed molecule generation algorithm RetroGEN help in the real-world drug discovery scenario to find potent inhibitors?***

The key goal in drug discovery is identifying potential inhibitors for real-world disease treatment or hit molecules that can be refined by medicinal chemists. This study focuses on assessing whether the molecules we generated show effective performance in wet-lab experiments.

***Question IV How does the generation process assist medicinal chemists in synthesis?***

In real-world synthesis, medicinal chemists work backward from the target molecule to identify the necessary precursor molecules or building blocks. This process helps them understand how to assemble these fragments into the desired molecule. In this work, we demonstrate how our fragment-based generation approach aids medicinal chemists in designing synthesis routes.

To answer the aforementioned questions, we conduct comprehensive experiments to prove their effectiveness and applications.

***Answer I***

To determine if the pretrained model has effectively incorporated the chemically relevant features, we fine-tune the parameters of the pretrained RetroCL model to perform a classification task on the derived USPTO-50K dataset. Specifically, given a chemical reaction, the altered chemical bonds during reactions are identified through atom mapping rules, and, subsequently, we combine the molecular graph with the altered bonds, as well as the randomly selected nonaltered bonds of the same molecular graphs to create the derived USPTO-50K dataset for classification in the fine-tuning stage. The USPTO-50K dataset has been divided into smaller subsets, each consisting of 1–40 k reactions respectively, enabling research into the efficacy of the pretrained chemical embeddings across varying dataset sizes for related chemical tasks.

To adapt the pretrained network to the classification of broken bonds in the downstream task, we introduce a prediction layer in the final layer of the model to predict whether the bonds will be broken in chemical reactions. The derived USPTO-50K dataset is split into training, validation, and testing sets in a ratio of 8:1:1. For fine-tuning the network, we use the Adam optimizer and set different learning rates for the pretrained model and prediction layer, specifically 1e-5 and 1e-4, respectively, with weight decay of 1e-4. We trained the model using a batch size of 32 and cross-entropy loss. We fine-tune the model for a total of 200 epochs and train it using 10 different seed values.

To validate the efficacy of the pretrained model, we compare it to a model that is fully supervised and trained on the derived USPTO-50K dataset and its subsets, which we will refer to as w/o RetroCL. The results of fine-tuning the model on the derived USPTO-50K and its subsets are presented in Table 1, as indicated by the AUC (area under the receiver operating characteristic (ROC) curve) metric.

**Table 1:** The testing AUC on USPTO-50K subset with different sizes between RetroCL and w/o RetroCL in the downstream task. Bold font represents the best-performing model. w/o RetroCL represents the supervised model without RetroCL as initialization.

Dataset Size	1 k	2 k	4 k	6 k	8 k	10 k	20 k	30 k	40 k	50 k
RetroCL	**0.841**	**0.860**	**0.873**	**0.872**	**0.883**	**0.879**	**0.889**	**0.895**	**0.898**	**0.900**
w/o RetroCL	0.835	0.857	0.863	0.867	0.878	0.873	0.885	0.893	0.893	0.896

As the results indicate, the pretrained RetroCL model applied in the bond classification task is slightly better than that of fully supervised approach in varying size subset of the revised USPTO-50K dataset. It can be concluded that the features acquired through contrastive learning can be seamlessly integrated into the bond classification fine-tuning task. This is because the objective of contrastive pretraining is to learn a representation that can distinguish between similar and dissimilar samples, which encourages the model to learn more robust and discriminative features that can be used for a wide range of downstream tasks. Besides, contrastive learning is a form of self-supervised learning strategy, where the model learns from the data without any explicit supervision, which allows the model to capture the structural differences to learn more diverse and complex features that may be difficult to capture in traditional supervised learning.

However, as can be seen above, the performance on the downstream task does not exhibit an obvious improvement as the size of dataset decreases. One possible reason for this phenomenon is that the bond classification task is relatively easy and both the model trained with and without pretrained RetroCL model can perform well. In this case, the representations may not provide a substantial improvement in the downstream task. Another possible reason could be due to inappropriate data augmentation in the field of molecular graphs. As is known to all, molecular graphs are bound by stringent rules and constraints. Some improper augmentations may lead to unrealistic or chemically implausible molecular graphs, which can cause the model to learn incorrect or misleading representations of the molecular data. In this case, there is an urgent need for researchers in the field of molecular science to develop more intrinsic molecular augmentations according to the knowledge of molecules to improve the performance.

**Figure 3:**
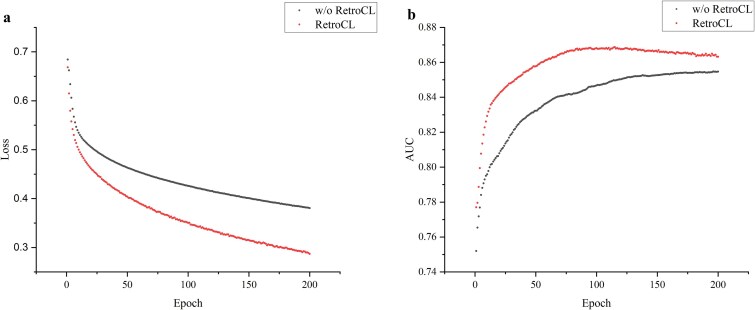
The comparison of RetroCL and w/o RetroCL on the training loss and testing AUC. (a) The loss curve during the training process. (b) The testing AUC of the RetroCL model and w/o RetroCL. w/o RetroCL represents the model trained without RetroCL initialization.

To further validate the effectiveness of the pretrained RetroCL model, the training loss on the bond classification task is visualized compared with the model that is trained without RetroCL as initialization. It can be observed that the loss of bond classification model with RetroCL is consistently lower than that without RetroCL initialization in Fig. 3a. Figure 3b illustrates the same phenomenon, where the model employing RetroCL as an initialization method outperforms the model without pretraining in terms of AUC on the test set. These observations indicate that the pretrained RetroCL model has acquired some useful features though it does not exhibit significant improvement. Given its effectiveness in the bond classification task, we will subsequently demonstrate its application in molecule generation.

***Answer II***

The primary goal of a molecular generation model is to produce molecules with the desired properties that make them viable as potential drug candidates. One of the most important features is validity, which is a key characteristic of a molecule that describes whether it meets the rules and principles of chemistry. Besides, we also expect our generation model can generate diverse molecules with distinct chemical structures and properties. Pharmacochemical filters are a crucial aspect of molecule evaluation by determining whether the generated molecules meet specific chemical rules and criteria. In this paper, we utilize the Glaxo [41], SureChEMBL [42], and PAINS [43] to assess the effectiveness of the generation model, which are widely recognized filters that identify molecules containing substructures known to cause issues in biological assays.

Table 2 demonstrates the performance of our generation model, RetroGEN, against ATR and CDK9 targets. Our model shows commendable performance compared with a number of well-established baseline models, notably MORLD and REINVENT, which are distinguished algorithms within the field of molecule generation. The RetroGEN model is capable of generating molecules with 100% validity and uniqueness against the ATR and CDK9 protein targets. In the case of the pharmacochemical filters for the ATR target, the RetroGEN model demonstrates superior performance over the baseline models in all three filters. Additionally, it exhibits a slightly improved performance in SureChEMBL and PAINS compared to its noninitialized counterpart. With respect to the CDK9 target, a similar trend is also observed, which can be concluded from Table 3. The RetroGEN model demonstrates slight improvements in the Glaxo and PAINS metrics, though it shows marginally lower performance in the SureChEMBL metric compared to REINVENT. Overall, the RetroGEN model could help in optimizing the properties related to pharmacochemical filters. RetroGEN’s high validity and uniqueness across ATR and CDK9 targets underscore its potential for generating diverse, drug-like molecules.

**Table 2:** The performance of RetroGEN on validity, uniqueness, Glaxo, SureChEMBL, and PAINS filters against ATR targets. Bold font represents the best-performing model. RetroGEN w/o represents the generation model without pretrained initialization.

	Glaxo↑	SureChEMBL↑	PAINS↑	Validity↑	Uniqueness↑
MORLD	0.607	0.046	0.727	**1.000**	0.842
REINVENT	0.685	0.420	0.848	0.879	0.9811
RetroGEN w/o	**0.764**	0.467	0.909	**1.000**	**1.000**
RetroGEN	0.711	**0.476**	**0.910**	**1.000**	**1.000**

**Table 3:** The performance of RetroGEN on validity, uniqueness, Glaxo, SureChEMBL, and PAINS filters against CDK9 targets. Bold font represents the best-performing model. RetroGEN w/o represents the generation model without pretrained initialization.

	Glaxo↑	SureChEMBL↑	PAINS↑	Validity↑	Uniqueness↑
MORLD	0.272	0.019	0.749	**1.000**	0.915
REINVENT	0.740	**0.593**	0.749	0.780	**1.000**
RetroGEN w/o	**0.806**	0.471	0.908	**1.000**	**1.000**
RetroGEN	0.784	0.483	**0.909**	**1.000**	**1.000**

The distribution of molecular properties is crucial for generating drug-like molecules in drug discovery. Key properties such as lipophilicity (LogP), synthetic accessibility (SA), drug-likeness (QED), and molecular weight (MW) are assessed to evaluate a molecule’s efficacy, safety, and similarity to existing drugs. We also use QuickVina 2 to assess binding affinity, which helps determine potential biological activity.

**Figure 4:**
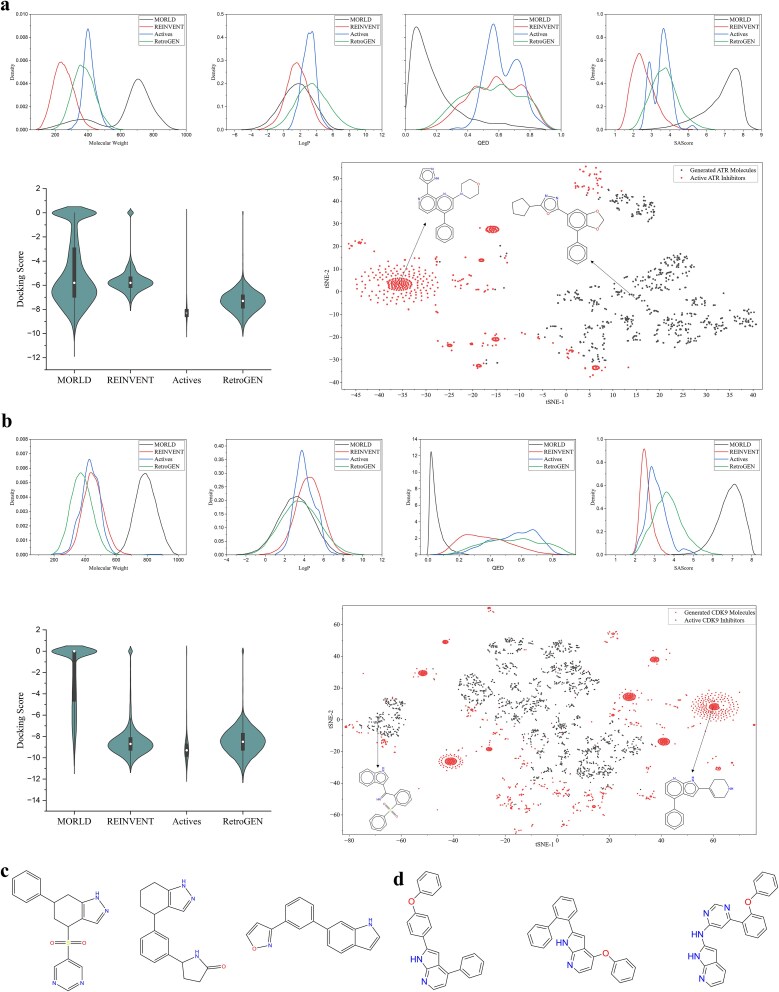
The property distribution analysis of generated ATR and CDK9 molecules, together with discovered novel scaffolds. (a, b) The top row displays the distributions of molecular weight, LogP, QED, and SAScore for molecules generated by different algorithms targeting ATR and CDK9, compared to active ATR inhibitors. RetroGEN closely matches the distributions of active molecules, while REINVENT and MORLD show varying degrees of alignment. In the second row, the left panel presents the distributions of docking scores, with RetroGEN showing the most stable performance on average. The right panel displays the T-SNE analysis of the generated molecule scaffolds compared to existing actives for the ATR and CDK9 targets, respectively, with a more widespread distribution compared to the existing actives. (c, d) The novel scaffolds of generated ATR and CDK9 inhibitors are shown, having been screened based on docking scores and similarity.

In Fig. 4a, we compare RetroGEN with MOLRD and REINVENT against the ATR target, alongside active molecules from BindingDB. The top row of Fig. 4a shows that the molecules generated by RetroGEN closely match the MW, LogP, QED, and SAScore distributions of active molecules for the ATR target, with REINVENT being the second closest in alignment. The molecular weight of molecules generated by RetroGEN predominantly falls within 250–500, aligning closely with active molecules. In contrast, REINVENT generates molecules with lower molecular weight, while MORLD generates larger molecules, which may hinder membrane permeability and absorption. For LogP, the distribution of existing Actives ranges from 0 to 6, with a concentration around 3. RetroGEN’s generated molecules also center around 3, with most falling between −2 and 5, similar to MORLD and REINVENT. For QED, RetroGEN and REINVENT perform similarly, with most generated molecules having a QED above 0.5. In contrast, MORLD’s generated molecules mostly have QED values below 0.5, indicating poorer performance. For SAScore, REINVENT outperforms other methods, likely due to the smaller molecular size of its generated molecules. As for docking performance, RetroGEN shows the best docking scores, indicating strong binding affinity. MORLD also achieves commendable scores, closely following RetroGEN, which suggests good binding potential. However, the larger molecular weight of MORLD’s compounds may violate the “Rule of Five” principle, making them less viable as drug candidates. Overall, RetroGEN exhibits the best performance and closely aligns with the active ATR molecules.

For the CDK9 target, as shown in Fig. 4b, RetroGEN aligns well with the distribution of existing potent CDK9 inhibitors, demonstrating a consistent trend. Regarding molecular weight, RetroGEN and REINVENT generate molecules with distributions similar to positive inhibitors, typically below 500, while MORLD produces molecules with larger molecular weights and a more varied distribution. For LogP, RetroGEN and MORLD exhibit similar distributions, both lower than the active compounds, while REINVENT aligns better with the desired properties, closely matching the LogP distribution of active CDK9 inhibitors. For QED scores, molecules generated by REINVENT tend to have lower QED scores, indicating lower drug-likeness. QED, calculated from descriptors like hydrogen bond donors, acceptors, and polar surface area, suggests that some properties may limit the molecules’ potential as preclinical candidates. In contrast, RetroGEN aligns well with existing inhibitors. In terms of SAScore, REINVENT performs best, with results closely matching existing inhibitors. RetroGEN has a slightly higher SAScore but still shows significant overlap with active compounds. MORLD’s larger molecular size increases synthetic complexity, with SASCore values primarily around 7. In docking scores, REINVENT performs exceptionally well, with RetroGEN closely following, both aligning closely with the active molecules. Overall, RetroGEN excels across nearly all metrics, with REINVENT also showing competent performance for both ATR and CDK9 targets. However, the key focus in molecule generation should not be solely on achieving the best scores in specific metrics, but rather on discovering novel and effective drug candidates.

Additionally, we explore whether our method can identify novel scaffolds. As shown on the right side of the second row in Fig. 4a, we compare the scaffolds generated for the ATR target with existing active inhibitors, using Bemis–Murcko scaffolds as the basis for comparison. The existing active ATR inhibitors exhibit a limited scaffold diversity, with many molecules sharing a common, focused scaffold. In contrast, the ATR molecules generated by RetroGEN display a broader variety of scaffolds, indicating greater structural diversity. A similar pattern is also observed in the distribution of generated CDK9 molecules and the existing CDK9 active inhibitors, as shown in Fig. 4b. Figure 4c and d showcases some of the novel scaffolds against ATR and CDK9 targets, respectively, identified through sequential docking screening and RDKit-based structural novelty evaluation, which could offer valuable insights to medicinal chemists.

***Answer III***

**Figure 5:**
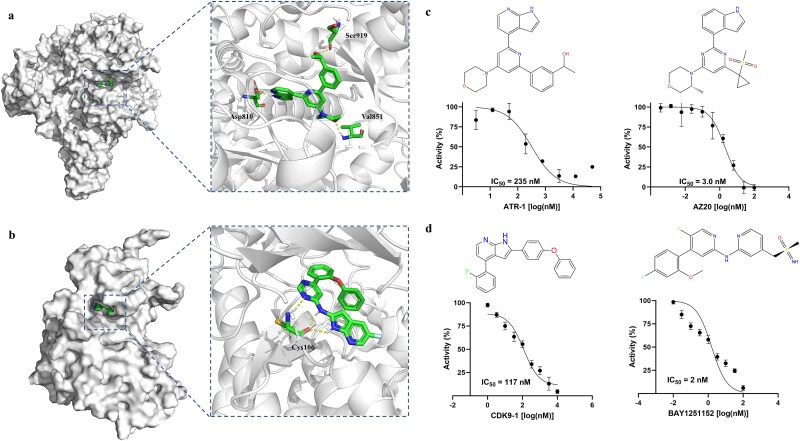
Docking pose analysis of generated inhibitors and corresponding dose–response curves for both generated and positive control inhibitors. (a, b) The docking poses of the generated ATR and CDK9 inhibitors, with yellow lines indicating hydrogen bond interactions between the ligands and their respective protein targets. (c, d) Dose–response curves showing the inhibitory effects of the generated compounds ATR-1 and CDK9-1, along with their respective positive control inhibitors, AZ20 for ATR and BAY1251152 for CDK9. The experiments were conducted in duplicate to ensure reliability.

To verify RetroGEN’s ability to identify novel inhibitors, we synthesized molecules generated for ATR and CDK9 based on their docking scores and similarity against its bioactive compounds collected from BindingDB, ensuring dual validation of efficacy and novelty. Their docking poses are checked for hydrogen bonds with the kinase hinge region, and molecules with novel scaffolds are selected for further testing. Figure 5a and b displays the docking poses of selected ATR and CDK9 inhibitors, visualized using PyMOL. Figure 5a shows that the residues Asp810, Val851, and Ser919 form hydrogen bonds with the ATR target, similar to highly active ATR inhibitors. In Fig. 5b, Cys106 of CDK9 forms multiple hydrogen bonds with the generated small molecule, contributing to its efficacy. To further evaluate the selected molecules, we synthesized and tested several compounds targeting ATR and CDK9, identifying ATR-1 and CDK9-1 as having notable inhibitory activity. The synthesis route for ATR-1 is outlined in Scheme S1 in the Supplementary Information, and its molecular structure was confirmed through ^1^H and ^13^C nuclear magnetic resonance (NMR) spectra, provided in Supplementary Fig. S1. These spectra verify the successful synthesis and purity of ATR-1, providing a solid foundation for subsequent biological evaluations. Similarly, the synthesis of CDK9-1 is detailed in Scheme S2 in the Supplementary Information, and its structure was characterized by ^1^H, ^19^F, and ^13^C NMR spectra (see Supplementary Fig. S2). These data confirm the molecular structure and the reliability of the synthesis of CDK9-1.

In Fig. 5c, the compound ATR-1 exhibits inhibitory activity against its target, with an IC_50_ value of 235 ± 26 nM, as shown on the left side of the figure. The dose–response curve illustrates the percentage of activity inhibition as the concentration of ATR-1 increases, following a typical sigmoidal curve. On the right side, the positive control, AZ20, demonstrates a significantly more potent inhibitory activity with an IC_50_ value of 3 ± 0.2 nM. Similarly, as shown in Fig. 5d, compound CDK9-1 demonstrates favorable inhibitory activity against CDK9, with an IC_50_ value of 117 ± 48 nM, as shown on the left side. As a positive control, BAY1251152 exhibits higher potency with an IC_50_ value of 2 ± 1 nM, as shown on the right. The comparison highlights the promising activity of CDK9-1, though it is less potent than the reference compound BAY1251152. The experiments were repeated twice to ensure the reliability of the results. Detailed methodology for the *in vitro* kinase assays is fully described in the Supplementary Information. Overall, these newly discovered inhibitors, ATR-1 and CDK9-1, demonstrate significant inhibitory activity, validating the robustness and reliability of the predictive models used. Although their potency may not be as high as the positive controls, these compounds can still inspire medicinal chemists and related researchers by offering novel scaffold solutions for further optimization and development. These findings provide a valuable starting point for exploring new chemical spaces and refining the activity of these inhibitors in future studies.

***Answer IV***

**Figure 6:**
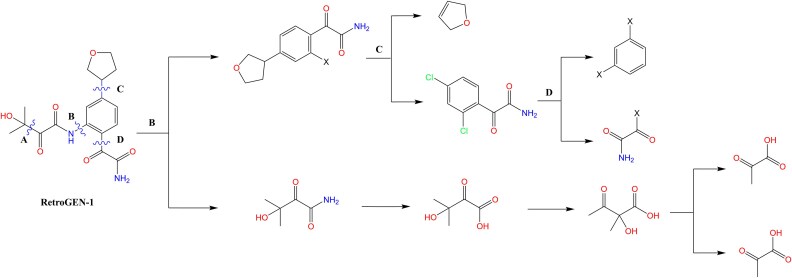
The comparison between the generated disconnection sites by RetroGEN and the predicted retrosynthesis routes from SciFinder. The figure shows the synthesis process of RetroGEN-1, with wavy lines indicating the newly formed bonds during generation. The right side presents the retrosynthesis routes of the compound RetroGEN-1 from SciFinder, highlighting the predicted breaking points (B, C, and D).

In this section, we will discuss how our model aids in retrosynthesis analysis. In the generation steps, the RetroGEN model operates by initially selecting a fragment from a fragment database, which is then attached to an existing scaffold to form a new chemical bond, with the bond formation strategy guided by reinforcement policy. Since the generation process is conducted fragment by fragment and the attachment strategy is refined through reinforced training, the fragment-based generation inherently incorporates retrosynthesis information. This allows the use of bond formation data as a prompt in the retrosynthesis process of molecules, providing valuable assistance to medicinal chemists. Figure 6 illustrates the process where a molecule begins with a benzene scaffold, and fragments are progressively attached to this existing scaffold until the model signals to halt, ultimately forming RetroGEN-1. The bonds formed during this generation process are marked with wavy lines. The right side of the figure presents the retrosynthesis analysis steps predicted by SciFinder (https://scifinder-n.cas.org), demonstrating a strong correlation between the bonds predicted to break (B, C, and D) and those created during the generation process. This alignment provides a valuable tool for medicinal chemists conducting retrosynthesis.

## Conclusion

In this study, we propose a novel bond augmentation method in contrastive learning, using retrosynthesis information to implicitly incorporate molecular properties during pretraining. The pretrained model is validated in identifying potentially broken bonds in retrosynthesis, demonstrating its ability to learn generalizable features and showing potential for related tasks. The fine-tuned model is later utilized in a reinforced generative model, providing a strong starting point for molecular state representation and policy initialization. Experimental results show that our model can generate novel molecules with desirable properties, aligning well with active molecules. Additionally, extensive wet-lab experiments confirm that the model successfully identifies potent inhibitors for specific protein targets.

**Key Points**

- We propose RetroCL, a novel chemically inspired bond augmentation method for contrastive pretraining, which demonstrates high performance in distinguishing broken bonds in chemical reactions.
- We employ contrastive pretraining in molecule generation through our model, RetroGEN, which demonstrates high performance across various molecular properties.
- The generation model RetroGEN effectively identifies novel drug candidates with potent kinase activity against ATR and CDK9, providing valuable insights for innovative drug discovery.

## Supplementary Material

Manuscript_Supplementary_Information_bbaf185

## Data Availability

The code is available at https://github.com/is-johnee/RetroGEN.
