# Supplementary material for: Self-awareness of retrosynthesis via chemically inspired contrastive learning for reinforced molecule generation
Source: Brief Bioinform. 2025 Apr 21;26(2):bbaf185. doi: 10.1093/bib/bbaf185 (PMC12009711; doi:10.1093/bib/bbaf185)
Supplement: Manuscript_Supplementary_Information_bbaf185 [file manuscript_supplementary_information_bbaf185.docx]

**Self-awareness of Retrosynthesis via Chemically Inspired Contrastive Learning for Reinforced Molecule Generation**

Yi Zhang^1,#^, Jindi Huang^1,#^, Xinze Li^1^, Wenqi Sun^2^, Nana Zhang^2^, Jiquan Zhang^2^, Tiegen Chen^3^ and Ling Wang^1,*^

^1^Guangdong Provincial Key Laboratory of Fermentation and Enzyme Engineering, Joint International Research Laboratory of Synthetic Biology and Medicine, Guangdong Provincial Engineering and Technology Research Center of Biopharmaceuticals, School of Biology and Biological Engineering, South China University of Technology, No. 382 Waihuan East Road, Higher Education Mega Center, Guangzhou 510006, China

^2^Guizhou Provincial Engineering Technology Research Center for Chemical Drug R&D, College of Pharmacy, Guizhou Medical University, No. 6 Ankang Avenue, Guian New District, Guiyang 561113, China

^3^Zhongshan Institute for Drug Discovery, Shanghai Institute of Materia Medica, Chinese Academy of Sciences, Zhongshan Life Science Park, No. 10 Heqing Road, Tsui Hang New District, Zhongshan 528400, China

^*^Corresponding author: Ling Wang, School of Biology and Biological Engineering, South China University of Technology, No. 382 Waihuan East Road, Higher Education Mega Center, Guangzhou 510006, China

E-mail: [lingwang@scut.edu.cn](mailto:lingwang@scut.edu.cn)

^#^These authors contributed equally to this work.

**Training details**

In this section, we will discuss the experimental specifics of both the pretraining and details regarding the reinforced generation process.

**Molecule Pretraining**

The USPTO-MIT dataset is utilized and reactions that have at least one altered bond are selected, while others are excluded. Besides, the duplicated reactions that are contained in the USPTO-50k dataset are removed. The open-source toolkit Indigo maps atoms within chemical reactions, tracing each atom's transitions and calculating the reaction's modifications based on the information about the changes observed at the atomic level. In cases where there are multiple bonds to be changed, graph augmentations and bond augmentations are applied randomly. However, if there is only one modified bond, graph augmentations are used. The features of chemical bonds are determined based on the local surroundings of its starting and ending atoms, taking into account a specific radius around them. 23 properties related to the local environment are used, and the details are explained in Supplementary Table 1. Of the local environment features, the number of nitrogen atoms, SP2 hybridization and aromatic bonds within the local environment are critical to the prediction of whether the bonds are disconnection sites in the retrosynthesis analysis. Molecular graph neural network is utilized for learning general representations of molecular structures, using a four-layer Graph Attention Network (GAT) with ReLU activation between each layer. The bond embedding model is implemented as a Multi-Layer Perceptron (MLP). The dimension for the graph embedding is set as 128, while the bond embedding is configured with a dimensionality of 32. A ratio of 0.2 is assigned in the augmentation process. We used mean operation for graph pooling. The graph and bond embeddings are concatenated and then projected through a nonlinear layer to get the final representations. We used the Adam optimizer with an initial learning rate of 0.001 and a batch size of 128 during the pretraining task. The model is trained for a fixed number of 40 epochs.

**Reinforced Molecule Generation**

**Protein Target** In this study, we employ Ataxia Telangiectasia and Rad3-related (ATR) and Cyclin-Dependent Kinase 9 (CDK9) kinases to demonstrate the capabilities of the model, which play a pivotal role in the molecular mechanisms of cell cycle regulation and transcriptional control. As there is no experimental high-resolution ATR structure, we use a rationally designed PI3K-alpha mutant (PDB ID 5UK8) which has been used in a wide variety of novel cancer therapeutics development and other disease treatments. The CDK9 is referenced with PDB ID 4BCI for molecule generation.

**Fragment Preparation** We employ the pre-collected universal fragments for generating target-specific molecules for CDK9 target, which has 91 unique fragments[1]. For the molecules generated for ATR target, we collected the active molecules against ATR from the publicly accessible BindingDB database, which provides experimentally determined binding affinities of protein-ligand complexes. To collect active molecules, those with biological activity values IC_50_ <= 100 nM are selected and the duplicates are removed. 80 fragments for ATR inhibitors are generated through CReM algorithm with radius 2 on the collected active datasets. We assess the generation capabilities against ATR and CDK9 targets using actives-specific and universal fragments respectively.

**Rewards Settings** In the reinforced generation stage, the rewards used are the docking score, which is calculated using QuickVina 2, a widely used and reliable molecular docking tool in computer-aided drug design. Additionally, we incorporate the Synthetic Accessibility Score (SAScore) and the Quantitative Estimation of Drug-likeness (QED) into the reward function to encourage the generated molecules that are not only effective in docking but also synthetically accessible and exhibit drug-like properties.

**General Settings** The configuration of the molecular graph and bond representation remains consistent with the parameters used in the pretraining phase. During the reinforced molecule generation process, the proximal policy optimization (PPO) algorithm is employed to optimize the molecular properties, with a learning rate set at 0.0001. In the generation process, the maximum step is set to 4, while the minimum step is established at 2. All the models are implemented using PyTorch and executed on a NVIDIA GeForce RTX 3080 Ti GPU.

**Supplementary Table 1. The features used in bond local information**[2]**.**

| **Feature Name** |
| --- |
| Fraction of SP2 |
| Fraction of SP3 |
| Fraction of other SP (not SP2 and SP3) |
| Number of Heteroatoms with Hs |
| Mean Atom Degree |
| Number of Aromatic Atoms |
| Number of Aromatic Bonds |
| Number of Atoms |
| Number of Bonds |
| Number of Conjugated Bonds |
| Number of Coupling Nitrogens |
| Number of Heteroatoms |
| Number of Homoatoms |
| Number of Nitrogens |
| Number of Nonaromatic Atoms |
| Number of Nonaromatic Bonds |
| Number of Nonconjugated Bonds |
| Number of Oxygens |
| Number of SP2 |
| Number of SP3 |
| Number of other SP (not SP2 and SP3) |
| Number of Coupling Nitrogens |
| Number of Sulfur |
| Number of Sulfur with no Oxygens |

**Target Compound Synthesis**

Chemical synthesis of the ATR-1 is depicted in Scheme S1. Starting from 2,6-dibromopyridin-4-amine (1), intermediate (2) was synthesized according to the literature. Intermediate (3) was prepared via regioselective Suzuki reaction. Treatment of compound (4) was prepared via Suzuki reaction. The target compound ATR-1 was obtained after the reduction reaction.

**Scheme S1. Synthesis of the target compound ATR-1**

Reagents and conditions: (a) NaOH, DMF, N2, 60℃, 5h; (b) Pd(PPh_3_)_4_, DME/H_2_O, K_2_CO_3_, 100℃, N_2_, 8 h; (c) Pd(PPh_3_)_4_, DME/H_2_O, K_2_CO_3_, 120℃, N_2_, 8 h; (d) LiAlH_4_, THF, N_2_, 45℃，24h.

Light yellow solid, 46% yield, mp 156℃. 1H NMR (400 MHz, DMSO-d6) δ 11.77 (1 H, s), 8.33 (1 H, d, J = 5.0 Hz), 8.16 (1 H, s), 8.06 (1 H, dt, J = 6.9, 1.9 Hz), 7.67 (1 H, d, J = 5.0), 7.61 – 7.54 (1 H, m), 7.45 (4 H, dd, J = 16.1, 8.8 Hz), 7.07 (1 H, dd, J = 3.4, 1.9 Hz), 5.23 (1 H, d, J = 4.2 Hz), 4.90 – 4.78 (1 H, m), 3.87 – 3.72 (4 H, m), 3.58 – 3.42 (4 H, m), 1.41 (3 H, d, J = 6.4Hz); 13C NMR (151 MHz, DMSO-d6) δ 157.36, 156.83, 150.21, 148.22, 143.02, 139.83, 128.74, 127.21, 125.60, 124.38, 117.75, 114.41, 106.51, 104.66, 101.18, 68.73, 66.34, 45.96, 26.49. HRMS (ESI): m/z [M+H]+ calcd. For [C24H25N4O2]+: 401.1978, found: 401.1989.


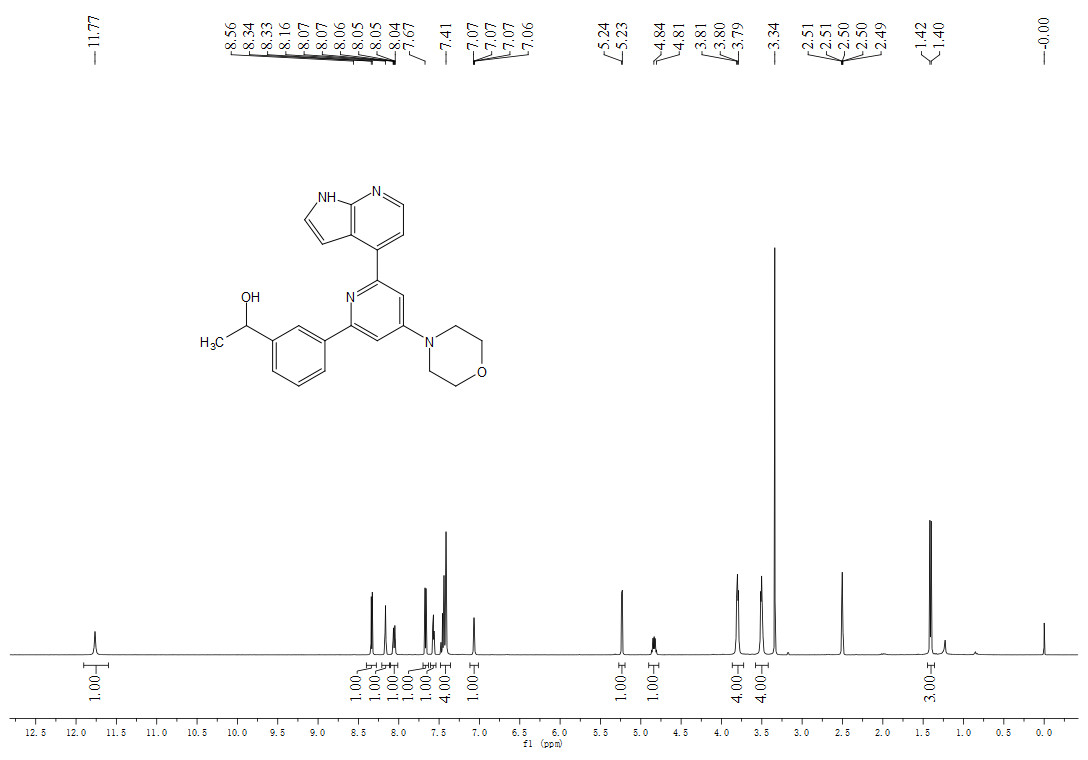


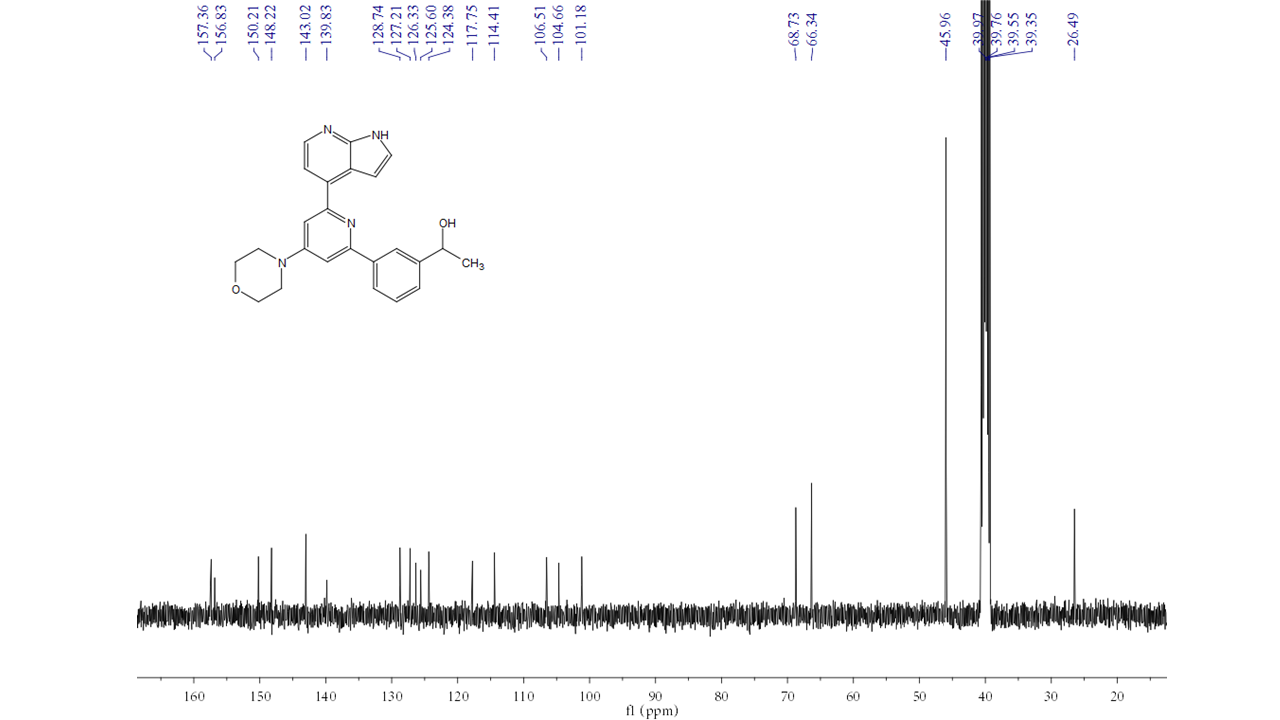


**Supplementary Figure 1. ^1^H and ^13^C NMR spectrum of the target compound ATR-1.**

Chemical synthesis of the CDK9-1 is depicted in Scheme S2. Compound 4-Chloro-1H-pyrrolo[2,3-b]pyridine undergoes amine protection using p-toluenesulfonyl chloride in the presence of NaH. The resulting protected intermediate is then treated with n-butyllithium to yield compound 1b. Subsequent dual Suzuki coupling reactions produce compound 1d. Finally, N-deprotection is achieved using NaOH, leading to the formation of the target compound, CDK9-1.

**Scheme S2. Synthesis of the target compound CDK9-1**

Reagents and conditions: (a) Tetrabutylammonium bromide, NaH, DCM, 0^o^C–r.t., 4.5 h. (b) (i) n-Butyllithium, diisopropylamine, THF, 0^o^C, 30 min; (ii) I_2_, THF, -78 – -10^o^C, 6 h. (c) Pd(PPh_3_)_4_, K_2_CO_3_, DME/H_2_O, 100^o^C, 11 h. (d) Pd(OAc)_2_, PPh_3_, Na_2_CO_3_, THF/H_2_O, 60 ^o^C, 10.5 h; (e) NaOH, 1,4-dioxane/H_2_O, 100^o^C, 4 h.

White powder, 80% yield, mp 230℃. ^1^H NMR (500 MHz, CDCl_3_) δ 12.10 (s, 1H), 8.34 (d, *J* = 4.8 Hz, 1H), 7.84 (d, *J* = 8.7 Hz, 2H), 7.67 (td, *J* = 7.5, 1.6 Hz, 1H), 7.49 – 7.42 (m, 1H), 7.39 (dd, *J* = 8.4, 7.5 Hz, 2H), 7.32 (td, *J* = 7.5, 1.0 Hz, 1H), 7.28 (d, *J* = 10.1 Hz, 1H), 7.21 (d, *J* = 4.5 Hz, 1H), 7.18 – 7.13 (m, 3H), 7.11 – 7.08 (m, 2H); ^19^F NMR (471 MHz, CDCl_3_) δ -114.66; ^13^C NMR (126 MHz, CDCl_3_) δ 160.89, 158.90, 157.83, 156.92, 150.33, 141.90, 139.55, 136.34, 131.45, 130.28, 130.21, 130.05, 127.49, 127.38, 124.54, 123.85, 119.37, 119.30, 117.01, 116.60, 116.42, 96.99. HRMS (ESI) *m/z*: [M+H]^+^ calcd*.* for [C_25_H_18_FN_2_O]^+^: 381.1398, found, 381.1398.


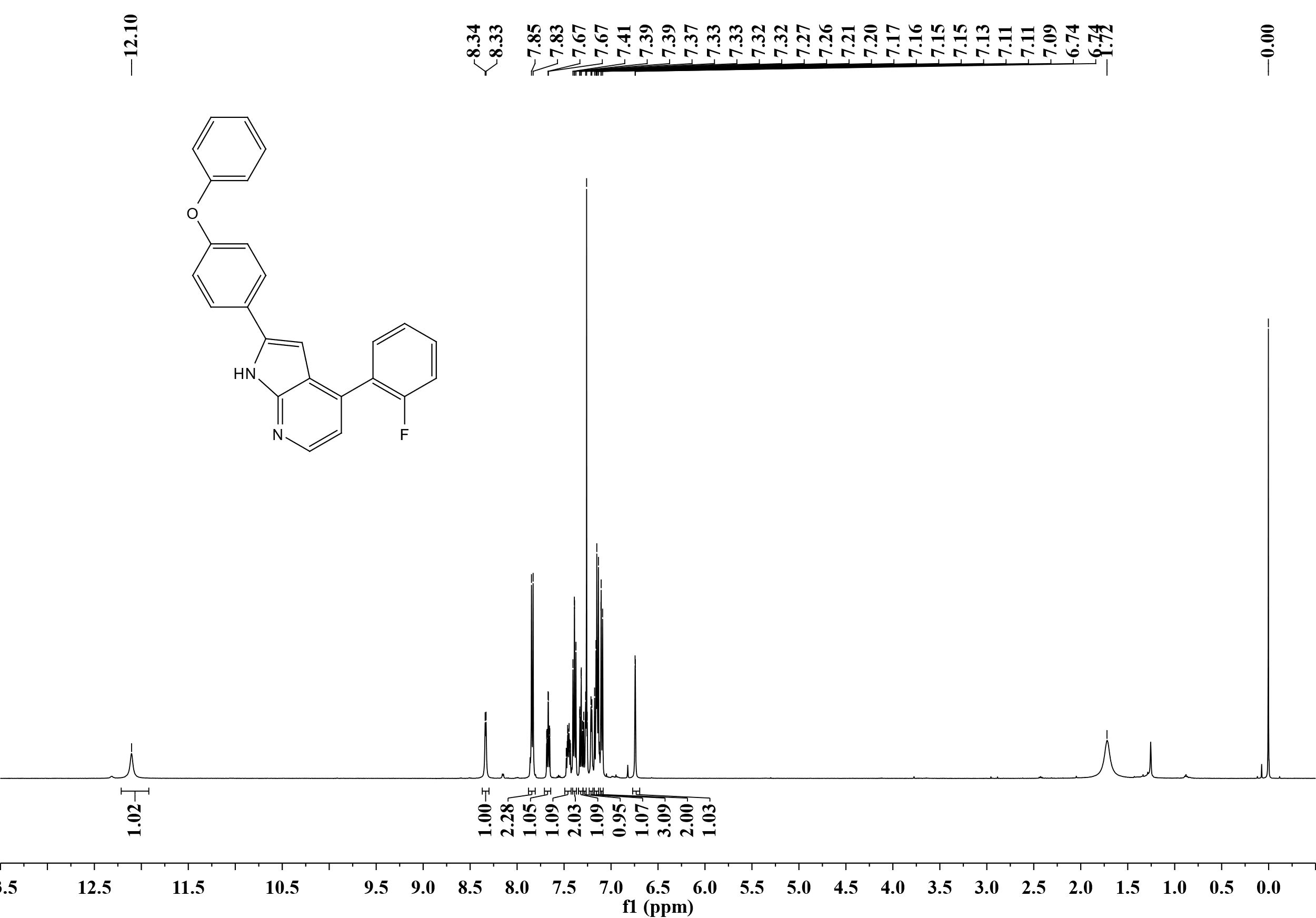


**
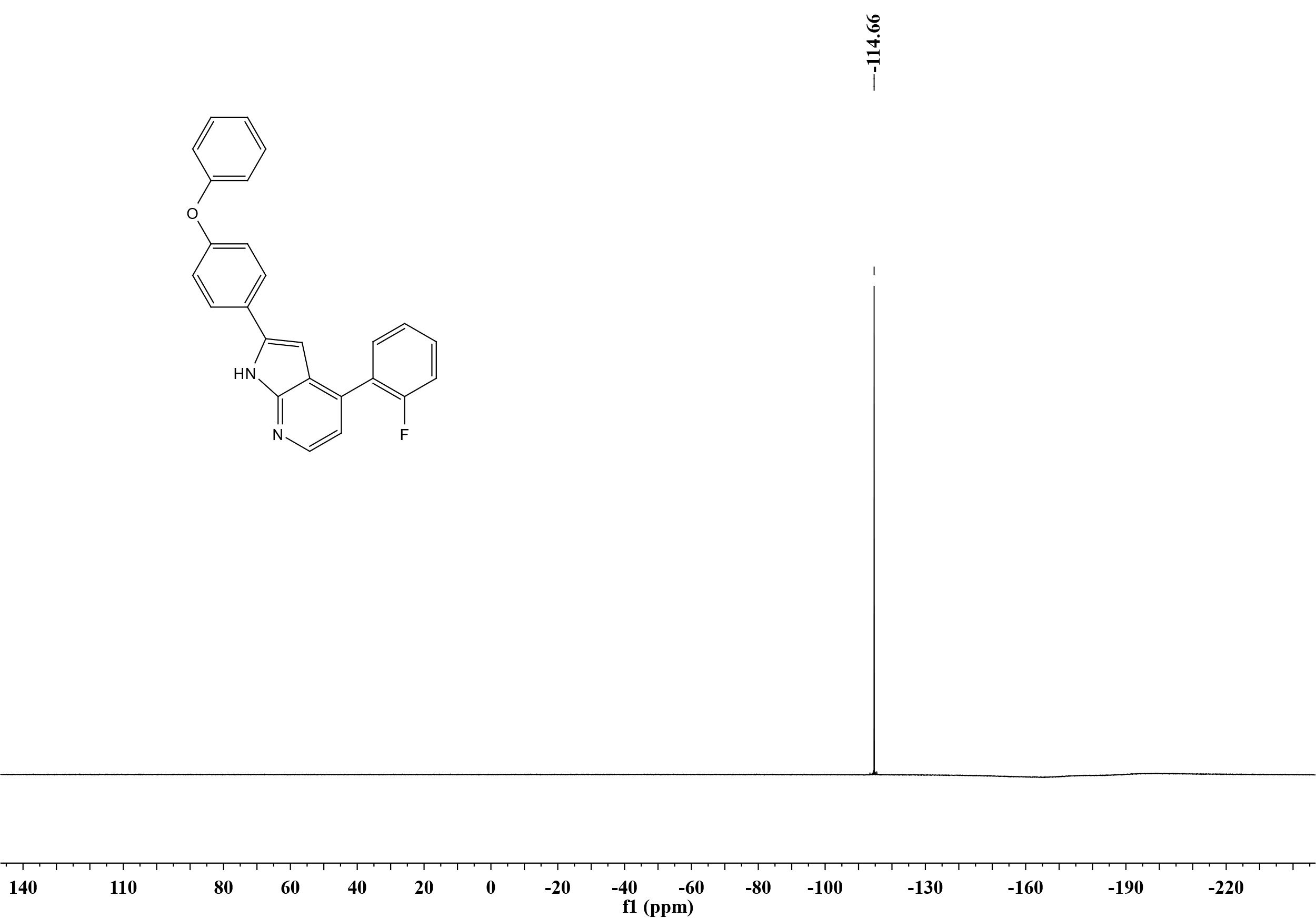
**

**
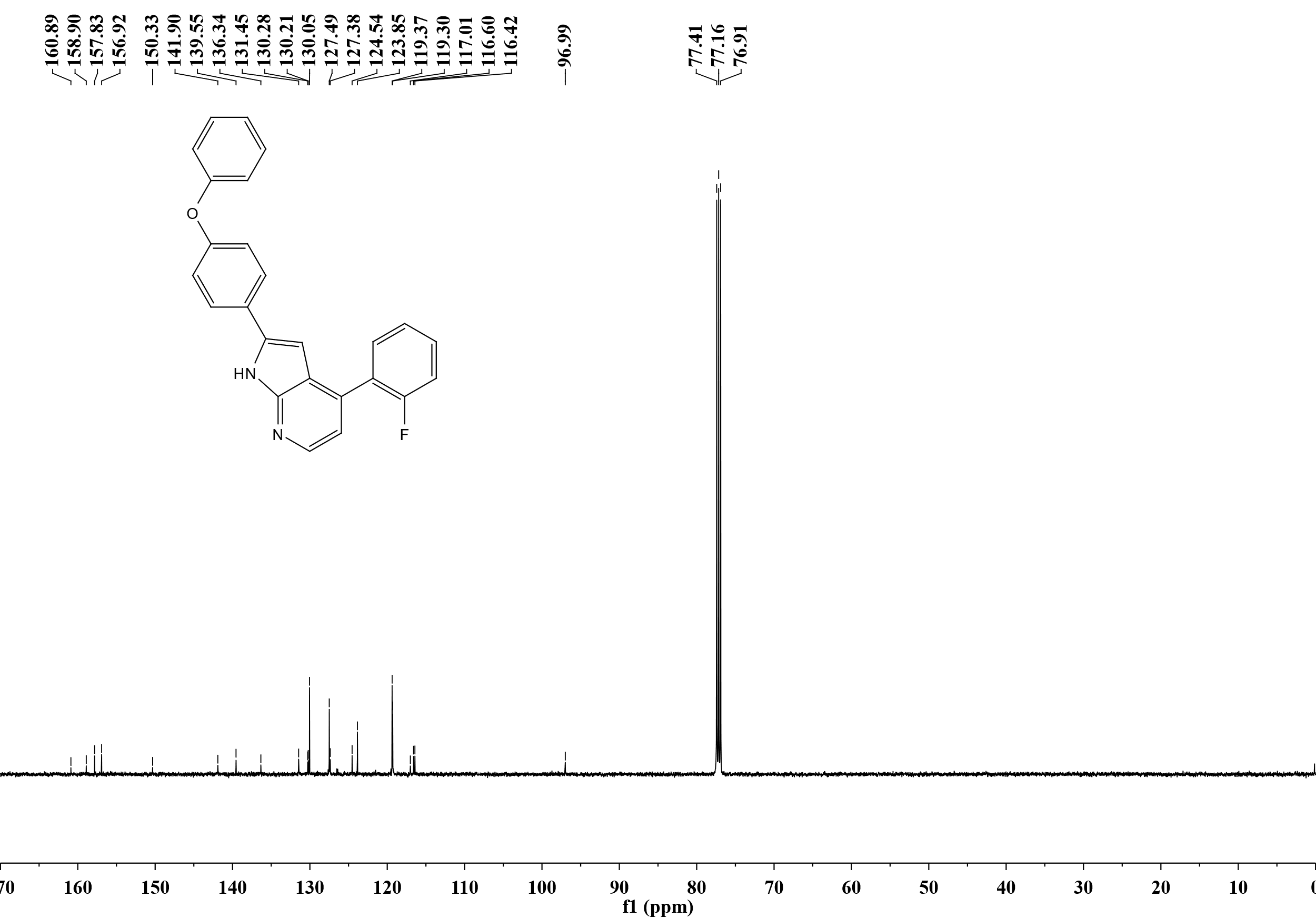
**

**Supplementary Figure 2. ^1^H, ^19^F and ^13^C NMR spectrum of the target compound CDK9-1.**

**In Vitro Kinase Assays**

The in vitro ATR kinase inhibition assay was performed by ChemPartner to evaluate the selected compound. All enzymatic reactions were conducted in a 384-well plate at 28℃. The assay used a 50 μl reaction mixture consisting of 1x kinase base buffer (50 mM HEPES, pH 7.5, 0.0015% Brij-35, and 1 M MnCl₂). The compound was diluted in 100% DMSO to the appropriate concentration, and 60 nl of the compound was transferred to the assay plate. A 2x ATR enzyme solution was prepared and added (10 μl) to each well, followed by a 10-minute preincubation. Next, 10 μl of a 2x FAM-labeled peptide and ATP solution was added to initiate the reaction. After incubation at 28℃ for the designated time, 30 μl of stop buffer (100 mM HEPES, pH 7.5, 0.015% Brij-35, 0.2% Coating Reagent #3, and 50 mM EDTA) was added to terminate the reaction. Data was collected using the Caliper system, and the conversion values were transformed into inhibition values using the formula: Percent inhibition = (max - conversion) / (max - min) * 100, where "max" represents the DMSO control, and "min" represents the low control. The data was then fitted using the XLFit Excel add-in (version 5.4.0.8) to obtain IC50 values.

In vitro CDK9 inhibition assay for the selected compound CDK9-1 was performed by Shandong Huawei Pharmaceutical Co. Ltd. All of the enzymatic reactions were conducted at 30℃ for 40 minutes. The 50µl reaction mixture contains 40 mM Tris, pH 7.4, 10 mM MgCl_2_, 0.1 mg/ml BSA, 1 mM DTT, 10 µM ATP, 0.2 µg/ml Kinase and 100 µM lipid substrates. The compounds were diluted in 10% DMSO and 5 µl of the dilution was added to a 50 µl reaction so that the final concentration of DMSO is 1% in all of reactions. The assay was performed using Kinase-Glo Plus luminescence kinase assay kit. It measures kinase activity by quantitating the amount of ATP remaining in solution following a kinase reaction. The luminescent signal from the assay is correlated with the amount of ATP present and is inversely correlated with the amount of kinase activity. The IC_50_ values were calculated using nonlinear regression with normalized dose−response fit using Prism GraphPad software.

**References**

1. Yang S, Hwang D, Lee S, et al. Hit and Lead Discovery with Explorative RL and Fragment-based Molecule Generation. Advances in Neural Information Processing Systems 2021; 34:7924–7936

2. https://github.com/Scienfitz/RDKit-reactive-site-features.
